# Histopathologic Finding of Both Gastric and Respiratory Epithelia in a Lingual Foregut Cyst

**DOI:** 10.1155/2015/278376

**Published:** 2015-07-30

**Authors:** Yangfan Luo, Nick Shillingford, Jeffrey A. Koempel

**Affiliations:** ^1^Keck School of Medicine of the University of Southern California, Los Angeles, CA 90033, USA; ^2^Department of Pathology, Children's Hospital Los Angeles, Los Angeles, CA 90027, USA; ^3^Division of Otolaryngology-Head and Neck Surgery, Children's Hospital Los Angeles, Los Angeles, CA 90027, USA

## Abstract

Foregut cysts are uncommon, mucosa-lined congenital lesions that may occur anywhere along the gastrointestinal or respiratory tract and typically present within the first year of life. Although infrequent, these cysts may generate feeding or respiratory difficulties depending on the size and location of the lesion. Foregut cysts of the oral cavity are rarely seen and of those cases localized to the tongue are even more uncommon. We describe a 4-month-old girl with a foregut cyst involving the floor of mouth and anterior tongue. Subsequent histologic analysis demonstrated a cyst lined with both gastric and respiratory epithelia. This case represents an extremely rare finding of both gastric and respiratory epithelia lined within a single cystic structure in the tongue. Although a very rare finding, a foregut cyst should be on the differential diagnosis of any lesion involving the floor of mouth or tongue in an infant or child.

## 1. Introduction

Cysts of foregut origin may localize anywhere along the gastrointestinal tract from the oral cavity to the anus although they are most commonly found in the ileum [1]. The vast majority of cases are seen in childhood, most of which are found in the first year of life. The presence of a lingual cyst often causes parents anxiety and may generate feeding or respiratory difficulties for the patient. Foregut cysts as a whole feature a wide variability of mucosal linings, typically involving gastric, squamous, intestinal, or respiratory epithelia or any combination of these epithelia. Reports of oral foregut cysts suggest that the most commonly observed epithelia are (in decreasing order) gastric, mixed gastric and squamous, intestinal, and mixed gastric and intestinal [2]. These cysts are generally uncommon but localization to the tongue and oral cavity is especially rare as this presentation comprises only 0.3% of cases [3]. The infrequent finding of a lingual foregut cyst likely reflects the particularly unusual circumstances required to generate a cyst of endodermal origin in the mouth and anterior tongue which are lined by ectoderm derived epithelia [4]. Given the aforementioned circumstances, the combination of a lingual cyst featuring both gastric and respiratory epithelia is remarkably rare; less than thirty cases in the literature are listed in Table 1 [5–23]. We describe our experience with a foregut cyst containing both gastric and respiratory epithelia in the tongue of a 4-month-old girl.

## 2. Case Report

**Figure 1:**
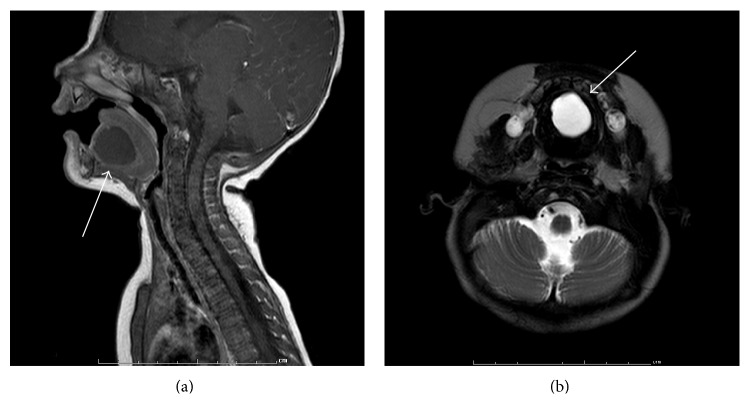
MRI. (a) T1-weighted, sagittal view: nonenhancing cystic mass in the sublingual space (arrow) without involvement of sublingual or submandibular glands. (b) T2-weighted, axial view: hyperintense mass midline in the anterior lower oral cavity (arrow).

An infant girl presented with a cystic mass on the floor of the mouth at 6 weeks of age. The slowly growing mass which was present at birth measured approximately 2 cm in diameter on initial examination. At the time of presentation, the patient weighed 4.075 kg, corresponding to the 11th percentile for her age. However, the patient showed no distress and the parents did not report any feeding or breathing difficulties. The family history was unremarkable and there was no history of any other anomalies. Two months later, a magnetic resonance imaging (MRI) was obtained and demonstrated a 1.8 × 2.0 × 1.9 cm T2 hyperintense and mildly T1 hyperintense nonenhancing cystic mass localized to the tongue and sublingual space in the midline with extension to the left of the midline. No fluid-fluid levels were noted. There was no indication that the cyst was arising from the sublingual or submandibular glands and no evidence of extension into the submandibular space (Figure 1). The differential diagnosis for this cystic mass included a dermoid cyst, an epidermoid cyst, a ranula, and, with lesser consideration given, a thyroglossal duct cyst and a lymphatic or venolymphatic malformation.

**Figure 2:**
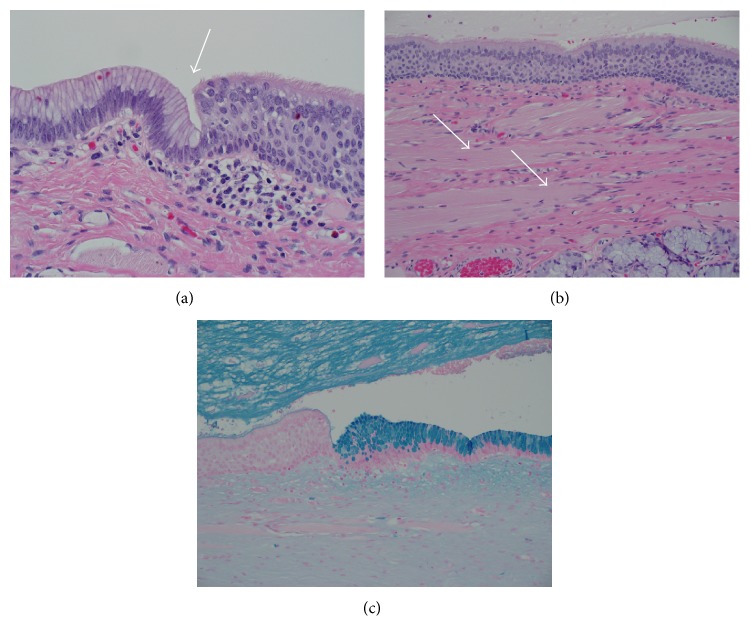
Histology. (a) Transition from respiratory-type ciliated pseudostratified columnar epithelium to gastric type epithelium (arrow). Hematoxylin and eosin, magnification at 400x. (b) The predominant epithelium of the cyst lining is respiratory-type ciliated pseudostratified columnar epithelium. Note the abundance of skeletal muscle with obvious cross striations in the cyst wall (arrows). Seromucinous glands are also present in the cyst wall (bottom right). Hematoxylin and eosin, magnification at 200x. (c) Alcian blue stain highlights the gastric epithelium while the adjacent respiratory-type epithelium is for the most part negative. Magnification at 200x.

Three weeks after the imaging study was performed, the cystic mass was resected in its entirety using bipolar electrocautery. The cyst was not opened during the procedure. Gross examination of the mass showed a cystic structure measuring 2.5 × 2.1 × 1.8 cm. Sectioning revealed a unilocular cyst measuring 2.5 cm at the greatest dimension which was filled with opaque gelatinous material. Microscopic sections showed a cyst which is lined by respiratory-type ciliated pseudostratified columnar epithelium punctuated at multiple sites by short segments of gastric epithelium. The cyst wall was composed of a continuous layer of skeletal muscle and, to a lesser extent, fibroconnective tissue. Foci of acute and chronic inflammation were present. Several seromucous glands were embedded in the skeletal muscle and fibroconnective tissue. The lumen was filled with mucinous material (Figure 2). Given these findings, a diagnosis of foregut cyst was made. The patient tolerated the procedure well with no apparent complications and no recurrence was observed on examination at 3-week follow-up.

## 3. Discussion

The pathogenesis of foregut cysts remains unclear owing to the variability of mucosal linings and locations across the reported cases. There is a general consensus that the often antenatal, neonatal, or infantile presentation of lingual cysts points to some early aberrant embryological event(s) leading to ectopic multipotent cells as the basis for cyst development. Even in the exceedingly rare adult cases, the examination and treatment typically arise from rapid recent changes in cysts observable since infancy or childhood [16, 19]. The observation of gastric, squamous, respiratory, or intestinal epithelium is consistent with cells of the foregut and suggests an endodermal origin. Perhaps the most widely accepted and frequently cited theory discussing foregut cyst formation was first elucidated by Veeneklaas in 1952. He proposed that derangement of notochord development and surrounding structures can lead to cyst formation due to endodermal cell entrapment or adherence during notochord plate infolding. The notion of notochord involvement derives from Veeneklaas' observation of an association between vertebral cleft and rib anomalies and intestinal duplications [24]. A more recent study by Qi et al. examined rat embryos with induced foregut duplications via adriamycin injection on gestational days 6–9. Their findings demonstrated that abnormal notochord development accompanied the occurrence of foregut cysts in fetuses exposed to adriamycin thus lending credence to the idea that notochord dysfunction may contribute to cyst formation [25]. The Veeneklaas theory can potentially be used to describe most, if not all, cyst development distal to the oral cavity which does include the majority of these cysts as a category but does not sufficiently encompass presentation of lingual cysts [3].

In 1970, Gorlin and Jirasek proposed that the heterotopic gastric mucosa seen in oral cysts may be the result of entrapped epithelium of the primitive stomach or stomatodeum [26]. Daley et al. would later supplement this theory with the notion that such epithelium would become entrapped between lateral swellings of the tongue around the 4th week of development and differentiate via inductive influences [27]. Subsequent histochemical studies of mucin found within cysts by Woolgar and Smith showed lectin staining patterns indicative of galactose,* N*-acetylgalactosamine, and galactosamine. These findings suggested a high degree of differentiation and varied from patterns seen in normal gastrointestinal epithelium, supporting an origin from undifferentiated endoderm subjected to inductive influence that could subsequently produce various cell types with varying degrees of differentiation [2, 11, 27]. Taken together, the elaborated Gorlin and Jirasek theory can explain the formation of cysts seen in the floor of the mouth and anterior tongue but fails to account for the presence of gastric and colonic cell types [28].

While a number of theories have been presented over the years, those proposed by Veeneklaas and Gorlin and Jirasek remain the most prominent. In any case, all explanations proposed thus far have failed to incorporate all locations and cell types described in the literature. These differences are readily apparent as the structural anomalies observed by Veeneklass and associated with intestinal foregut cysts, or any other abnormalities for that matter, are notably absent in most reports of lingual foregut cysts. Indeed, the variety of presentations suggests a number of different, albeit possibly similar, causes leading to the manifestation of the various clinical entities classified as foregut cysts or choristomas. It may therefore be impossible to reconcile the development of all cysts under a single theory.

A variety of designations have been used to refer to these cysts in the literature including “lingual cyst,” “enteric duplication cyst,” “foregut cyst,” “lingual choristoma,” “gastrointestinal cyst of the tongue,” and “lingual alimentary cyst,” as well as combinations of the aforementioned terms. Given the confusion surrounding the nomenclature, the use of histologic descriptive terms endorsed by Manor et al. should be favored until these pathologies are better understood or distinguished [29]. While the term “foregut cyst” is not explicitly used by Manor et al., we believe it is appropriate here given the epithelia observed and the typical age of presentation which heavily implicate the developmental origin and thus serve as an additional descriptor. Furthermore, it should be noted that duplications refer to a specific type of cyst characterized by (1) a coat of smooth muscle, (2) attachment to a part of the alimentary tract, and (3) a mucosal lining similar to that of some part of the alimentary tract although not necessarily the part to which it is attached [6]. As such, we believe that “duplication” may be a suitable label provided that such cases are understood to satisfy all three criteria, thus further distinguishing them by virtue of a smooth muscle coat. Consequently, the lesion in this report is described as a lingual foregut cyst with gastric and respiratory epithelium.

Review of reports of lingual cysts involving both gastric and respiratory epithelia suggests a male predilection (Table 1) which has also been observed to varying degrees in reviews of oral cysts in general [2, 4, 30]. Most reported cysts present during the first year of life and localize to the anterior tongue and/or the floor of the mouth. In recent years, modern imaging modalities have increasingly allowed for antenatal discovery of cysts. This provides healthcare practitioners with the opportunity to anticipate and prepare for any complications such as obstruction of the upper airway during or immediately after delivery [17, 18, 20, 23].

Patients are usually asymptomatic at the time of identification. Among the roughly one-third of cases reporting symptoms, at least half involved some period of observation following discovery and prior to presentation with complaints. Feeding and respiratory difficulties are usually the greatest cause of concern for lingual foregut cysts given the very young age of most patients at the time of presentation but the obstruction or mass effect produced by a cyst can also result in dysphagia, dysphonia, and pain or discomfort [11, 15–17, 19, 21–23].

CT or MRI is often useful in defining the dimensions and anatomic involvement of a cyst and assists in preoperative planning for excision but the definitive diagnosis usually cannot be confirmed until the histopathologic analysis of the specimen. Surgery should be considered, even in the absence of symptoms or complaints, as evolution to malignancy in adults has been reported in previously untreated cysts [16, 19]. Excision is also supported by the possibility of continued growth leading to obstruction, perforation, or infection. Furthermore, the relatively more common gastric mucosa and glands seen in some foregut cysts can theoretically lead to ulceration and bleeding. Although not reported in any cases of lingual cysts, such complications have been described in foregut cysts and other duplications elsewhere along the alimentary tract [31–33]. Needle aspiration may provide temporary relief, particularly in the setting of airway obstruction, but does not represent a definitive measure given the propensity for recurrence [30]. Complete surgical excision is highly recommended as it is generally curative and recurrence is rare. A foregut cyst should be on the differential diagnosis of any cystic mass in the oral cavity in an infant or a young child.

## Figures and Tables

**Table 1:** Reported cases of intraoral cysts involving gastric and respiratory epithelia.

Author (year)	Sex	Age	Location	Presentation	Notes
Shioda et al. (1971) [5]	—	—	FOM	—	

Brown and Kerr-Wilson (1978) [6]	M	11 mo.	Ventral surface of tongue	Asymptomatic	

Tschen (1978) [7]	M	2 yr.	FOM	Asymptomatic	

Mirchandani et al. (1989) [8]	M	6 mo.	Ventral surface of tongue	Asymptomatic	

Mir et al. (1992) [9]	M	5 mo.	Left FOM + dorsum of base of tongue	Asymptomatic	2 cysts in patient

Ohbayashi et al. (1997) [10]	M	11 mo.	Dorsum of tongue	Asymptomatic	

Said-Al-Naief et al. (1999) [11]	F M	2 yr. 2 mo.	Anterior tongue Anterior FOM	Difficulty in eating and speech Asymptomatic	

Mandell et al. (2002) [12]	F	14 days	Ventral surface of tongue	Asymptomatic	

Noorchashm et al. (2004) [13]	—	Neonate	Anterior FOM	Asymptomatic	Featuring an intraosseous component through mandible

Hall et al. (2005) [14]	M	13 days	Base of tongue + FOM	Asymptomatic	

Leung et al. (2007) [15]	F	2 days	FOM	Difficulty in feeding	

Agaimy et al. (2007) [16]	M	41 yr.	FOM	Dysphagia, dysphonia	Present at birth; recent malignancy

Hartnick et al. (2009) [17]	F	10 days	FOM	Obstruction of larynx	

Houshmand et al. (2011) [18]	F F	2 days 2 mo.	Anterior tongue Anterior tongue	Asymptomatic Asymptomatic	

Patel et al. (2011) [19]	M	45 yr.	Anterior tongue	Dysphagia, severe pain, and impaired speech	Present since childhood; recent malignancy

Blanchard et al. (2012) [20]	M M	Neonate Neonate	Anterior tongue Ventral surface of tongue	Asymptomatic Asymptomatic	

Joshi et al. (2013) [21]	M	28 mo.	Ventral surface of tongue	Initially asymptomatic, possible mechanical effect delayed speech	

Pentenero et al. (2013) [22]	M	15 yr.	Anterior tongue + FOM	Progressive dysphagia, dysphonia	Slow growth over 5 years

Gantwerker et al. (2014) [23]	F	Neonate	Anterior tongue	Respiratory difficulty	

Present case	F	4 mo.	Ventral surface of tongue + FOM	Asymptomatic	

FOM: floor of mouth.
